# Genome-streamlined SAR202 bacteria are widely present and active in the euphotic ocean

**DOI:** 10.1093/ismejo/wraf049

**Published:** 2025-04-08

**Authors:** Changfei He, Michael Gonsior, Jihua Liu, Nianzhi Jiao, Feng Chen

**Affiliations:** State Key Laboratory of Marine Environmental Science, College of Ocean and Earth Sciences, Carbon Neutral Innovation Research Center and Fujian Key Laboratory of Marine Carbon Sequestration, Xiamen University, Xiamen 361102, PR China; Institute of Marine and Environmental Technology, University of Maryland Center for Environmental Science, Baltimore, MD 21202, United States; Chesapeake Biological Laboratory, University of Maryland Center for Environmental Science, Solomons, MD 20685, United States; Institute of Marine Science and Technology, Shandong University, Qingdao 266237, China; State Key Laboratory of Marine Environmental Science, College of Ocean and Earth Sciences, Carbon Neutral Innovation Research Center and Fujian Key Laboratory of Marine Carbon Sequestration, Xiamen University, Xiamen 361102, PR China; Institute of Marine and Environmental Technology, University of Maryland Center for Environmental Science, Baltimore, MD 21202, United States

**Keywords:** Sar202, small genome size, low GC, genome reduction, euphotic ocean

## Abstract

SAR202 bacteria are a diverse group of bacteria in the ocean. The SAR202 lineages dominate the bacterial community and evolve specialized metabolisms for oxidizing recalcitrant organic compounds in the dark ocean. SAR202 bacteria are also present in the euphotic oceans; however, their ecological roles and metabolic potential remain poorly understood. In this study, we collected 392 non-redundant metagenome-assembled genomes from different oceans, with 18% of these SAR202 genomes characterized by small genome sizes (<2 Mbp), low GC content (<40%), and high gene density. The 70 genome-streamlined SAR202 bacteria constitute more than an average of 90% of SAR202 in the euphotic zone and exhibit streamlined metabolic features compared to the dark ocean SAR202. Genome-streamlined SAR202 is distributed in many major SAR202 lineages (i.e. I, II, III, and VI). Phylogenomic analysis shows that the genome-streamlined SAR202 clades diverged from the non-genome-streamlined SAR202 lineages and evolved independently within the same clades. Certain genes are enriched in genome-streamlined SAR202, such as proteorhodopsin genes and the coding genes of major facilitator superfamily transporters, nucleoside transporters, and deoxyribodipyrimidine photo-lyase, indicating their adaptation to sunlit oligotrophic water. A detailed comparison between genome-streamlined SAR202 and non-genome-streamlined SAR202 was made to illustrate their distinct niche distribution and metabolic buildup. In addition, the metatranscriptomic analysis supports that genome-streamlined SAR202 bacteria are active in the upper ocean. This study represents a systematic study of streamlined SAR202 bacteria that occupy the euphotic ocean and provides a comprehensive view of the ecological roles of SAR202 bacteria in the ocean.

## Introduction

SAR202 bacteria, belonging to the phylum *Chloroflexota*, were identified in the ocean through 16S rRNA gene sequencing [1, 2]. SAR202 bacteria are ubiquitous and abundant in the ocean [3–8]. Since 2017, the importance of SAR202 bacteria has been recognized due to their potential role in degrading recalcitrant dissolved organic matter (RDOM), which makes up the largest organic carbon inventory in the dark ocean [4, 8–10]. They are among the most active bacterial groups in the deep ocean as reflected by the activities of key genes involved in the degradation of RDOM [11, 12]. A few closely related strains of SAR202 were successfully cultivated recently [13]; however, SAR202 still lacks many cultured representatives from other major groups and subgroups such as SAR202 group II, group III, and subgroup Ic. In the past several years, single-cell genomics, metagenomics, and metatranscriptomics have been widely used to explore the diversity, distribution, genomic features, and gene expression of SAR202 in the ocean [4, 6, 8–10, 12, 14, 15]. SAR202 bacteria are genetically diverse and contain seven major groups [4] and >20 subgroups [9]. The distribution of SAR202 in the water column shows a clear vertical niche adaptation of SAR202 subgroups, suggesting a potential functional difference among SAR202 genotypes likely due to niche adaptation [4, 8, 9].

Genomic properties such as genome size, Guanine-Cytosine (GC) content, coding density, number of genes, and gene synteny can provide insight into microbial ecological strategies. For example, the adaptation to oligotrophic surface water of SAR11 bacteria is reflected by their streamlined genome sizes (1.3–1.4 Mbp), low GC content (~30%), and high coding density [16–19]. For SAR202, many metagenome-assembled genomes (MAGs) have been reported [4, 8–10, 15]. Some earlier studies reported different ranges of SAR202 genome sizes based on a limited number of MAGs and low sequencing depths [8, 10, 11, 15], which resulted in an incomplete estimate of SAR202 genome size and other genomic properties. Moreover, the number of MAGs is not evenly distributed among SAR202 groups and subgroups. Certain abundant SAR202 groups (i.e. group III) have more MAGs and higher MAG quality than the less abundant SAR202 groups. In addition to the low relative abundance of SAR202, the complex microbial community in the upper ocean also leads to poor recovery of SAR202 MAGs from the photic zone [9]. In general, a comprehensive understanding of SAR202 genomic properties is still lacking, especially in the upper ocean.

Recently, we used deep metagenomic sequencing (180 Gbp per sample) to explore microbial genomes in six major depths from 4 to 4535 m at the Bermuda Atlantic Time Series (BATS) site and recovered 173 SAR202 MAGs from these six samples [9]. Our MAGs covered all seven major groups of SAR202 and added 12 new subgroups to the current SAR202 cluster. Together with other ocean metagenomes, we illustrated an updated depth profile of 20 SAR202 subgroups. Using high-quality MAGs with over 90% completeness, we compared the metabolic pathways across SAR202 subgroups based on the near-complete genomic properties of SAR202 bacteria.

Here, based on the currently known SAR202 genomes, we reported the prevalence of genome-streamlined SAR202 (gsSAR202) in the upper ocean. These gsSAR202 were defined based on their small genome size (<2 Mbp), low GC content (<40%), and low ratios of intergenic spacer DNA to coding DNA [20]. gsSAR202 bacteria occupy the photic zone, carry the proteorhodopsin (PR) gene, and evolve independently within several major SAR202 groups. On average, 90% of the SAR202 community in the euphoric ocean consisted of gsSAR202. The comparison of metabolic genes between gsSAR202 and non-gsSAR202 revealed the reduction of genes responsible for carbohydrate and aromatic degradation in gsSAR202.

## Materials and methods

### Collection of SAR202 genomes

The SAR202 genomes analyzed in this study were obtained from our previous work [9], including samples from the Tara Ocean, marine trenches, Malaspina, and others. A total of 471 SAR202 genomes were retrieved, including 173 MAGs from deep metagenome sequencing at the BATS site [9]. The BATS water column samples were collected at six different depths (4, 106, 805, 2000, 2373, and 4535 m) using Niskin bottles aboard the R/V Atlantic Explorer on 5–11 August 2019, station information is shown in Supplementary Fig. S1. Environmental factors were recorded by CTD sensor. Metagenome binning followed methods described in our previous study [9]. Specifically, we used MEGAHIT v1.2.9 [21] to assemble clean sequences, retaining assembled contigs >2000 bp. These contigs were automatically binned into MAGs using Metabat2 v2.12.1 [22], Maxbin2 v2.2.4 [23], and CONCOCT v1.0.0 [24]. All SAR202 genomes were dereplicated using dRep [25] with an ANI cutoff of ≥95%, resulting in 392 nonredundant SAR202 MAGs, which were used for this study (Supplementary Table S1). The completeness and contamination of the SAR202 bacteria were evaluated by CheckM v.1.0.7 and BUSCO v4 [26, 27]. These SAR202 genomes were classified using the GTDB-Tk tool [28]. The naming of SAR202 groups and subgroups follows a recent paper [9].

### Metagenome fragment recruitment analyses

For calculating the relative abundance of SAR202 bacteria, the metagenomic samples used for fragment recruitment included 6 different depth samples from BATS [9], 75 samples from Tara Oceans [29], and 26 samples from the Malaspina 2010 expedition (http://www.expedicionmalaspina.es) [30]. These samples were collected from the different typical water layers in the world ocean, including surface ocean (SRF, 0–5 m depth), deep chlorophyll maximum (DCM, 25–250 m depth), mesopelagic ocean (250–1000 m depth), and bathypelagic ocean (>1000 m depth) (Supplementary Table S2). Salmon v 0.13.1 [31] was applied to calculate the coverage of all SAR202 contigs in each sample. Coverage tables were acquired to assess the Transcripts Per Million (TPM) abundance of each contig in each sample. The TPM abundance of each bin in each sample was calculated by taking the length-weighted average of the contig abundances with script split_salmon_out_into_bins.py in the metawrap [32].

### Phylogenomic tree reconstruction

All representative *Dehalococcoidia* genomes of the GTDB database (*n* = 70) were used as outgroups. A SAR202 bacteria phylogenetic tree was built using 120 core genes which were identified using GTDB-Tk v0.1.3 [28]. These core genes were aligned and concatenated using the gtdbtk align modules with default parameters. If a genome has a low number of markers identified, it will be excluded from the analysis at this step. Iqtree [33] was used to analyze the aligned sequences and construct the phylogenomic tree. We employed the ModelFinder feature within iqtree to determine the best-fit substitution model, followed by tree reconstruction with 1000 ultrafast bootstrap replicates to assess branch support. Branches with bootstrap values greater than 50 were considered reliable. The SAR202 phylogenetic tree was visualized using iTOL [34].

To construct the 16S rRNA gene phylogenetic tree, RNAmmer 1.2 software [35] was applied to extract the 16S rRNA gene sequences from all SAR202 genomes. *Dehalococcoides ethenogenes* (AF004928) served as an outgroup. The 16S rRNA gene sequences were aligned using MAFFT version 7.4 [36] to ensure accurate alignment. The aligned sequences were input into iqtree software version 2.1.3 [33] for phylogenetic analysis. Phylogenetic tree reconstruction was conducted with 1000 approximate likelihood-ratio test replicates to assess branch support. Branches with bootstrap values greater than 50 were considered reliable. The 16S rRNA gene phylogenetic tree was visualized using iTOL [34].

To estimate the divergence times of SAR202 bacteria, we utilized r8s (v1.7) to calibrate a phylogenetic tree constructed from aligned 120 core genes of 98 high-quality SAR202 [37]. The sequences were aligned using MAFFT version 7.4 [36], and a maximum likelihood phylogenetic tree was inferred using iqtree version 2.1.3 [33] with the GTR model. The raw divergence time tree file is available as Zenodo. The order *Chloroflexales* (*Chloroflexus aggregans* and *Roseiflexus castenholzii*) at 651.5 million years ago (MYA) and *Dehalococcoidales* (*Dehalococcoides mccartyi* and *Dehalogenimonas formicexedens*) at 496 MYA were used as two outgroups for calibration [38].

### Gene prediction, annotation, and streamlining characterization of SAR202 bacteria

All medium-high quality SAR202 genomes (>50% completeness) were used for genomic analysis. Open reading frames (ORFs) were predicted using Prokka v1.14.63 [39], and the resulting genes were annotated against the Kyoto Encyclopedia of Genes and Genomes (KEGG) [40] and eggNOG databases [41] using DIAMOND v0.9.14, with an E-value threshold of 1 × 10^−6^ [42]. Coding sequences (CDS) and tRNA genes were quantified from the gff files of each genome.

To investigate the genomic properties of the medium/high-quality SAR202 genomes, the estimated genome size was calculated by dividing the MAG’s assembly size by CheckM completeness (ranging from 0 to 1) [43]. We applied the concept of richness to evaluate gene family diversity in the SAR202 bacteria. Gene richness represents the proportion of gene families in a specific SAR202 MAG relative to the total gene families across all SAR202 MAGs, with higher values indicating greater metabolic capacity. Additionally, the coding proportion within each genome was calculated by dividing the base pairs of coding regions by the MAG’s assembly size.

### Transcriptional analysis of gsSAR202 in the euphotic zone

Transcriptional data from the Tara Ocean project were downloaded to analyze the expression of gsSAR202 in the surface ocean, with sample details provided in Supplementary Table S3. Low-quality reads were removed from the raw transcriptional data using Trimmomatic v0.36 [44]. The predicted gsSAR202 genes were used as the reference gene set for metatranscriptomic analysis. An index for the reference gene set was built using Salmon v0.13.1, and transcript abundance was estimated via quasi-mapping during quantification [31].

### PR gene analysis of SAR202 bacteria

To identify the PR genes, the ORFs of SAR202 genomes were annotated against the KEGG and eggNOG databases with diamond (E-value threshold of 1 × 10^−6^). We downloaded 694 rhodopsin gene sequences from a previous study [45]. Multiple alignments were performed using mafft [36], and the maximum likelihood tree was constructed based on the aligned PR sequences with iqtree via 1000 iterations [33].

### Statistics analysis

**Figure 1:**
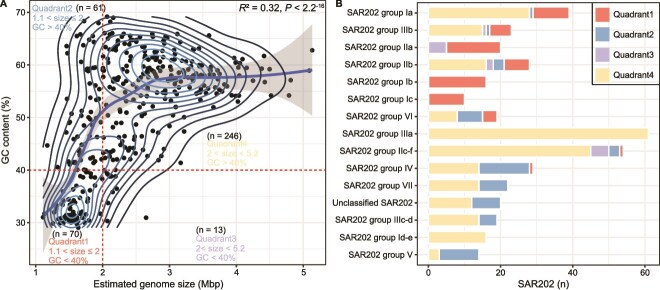
Correlations and distributions of GC content and genomic size in SAR202 bacteria. The relationship between GC content and genomic size across SAR202 species (A) is divided into four quadrants based on these metrics. A line represents the linear regression model. Distribution of SAR202 bacteria across the defined quadrants, organized by SAR202 groups and subgroups (B).

All analyses and visualizations were conducted using R (version 3.6.1). Linear regression analyses were performed with the geom_smooth function from the ggplot2 package [46]. A linear regression analysis revealed a significant increase in GC content among genomes ranging from 1.1 to 2 Mbp, followed by a plateau in genomes exceeding 2 Mbp (Fig. 1A). Establishing thresholds at a genome size of 2 Mbp and a GC content of 40%, SAR202 bacteria can be systematically categorized into four quadrants. Bar and dot charts were also generated using ggplot2 [46]. The pie charts were created with the Plotrix package [47]. Heatmaps were produced using the pheatmap package [48]. To investigate the relationships between small genome size SAR202 and environmental parameters, we analyzed data from Tara Ocean station samples from the surface and DCM zones. Environmental parameters were sourced from a previous study [49]. We calculated Spearman’s rank correlation coefficients between 70 small genome SAR202 and 42 environmental factors using the WGCNA package [50]. Positive correlation (*r* > 0.3, *P* < .05) and negative correlation (*r* < −0.3, *P* < .05) network were constructed using the igraph package [51]. The edgeR package [52] was used to analyze the significantly different genes between gsSAR202 and non-gsSAR202 genomes, with significance determined by |logFC| > 2 and *P* < .05.

## Results and discussion

### Genome streamlining of SAR202

Analysis of 392 nonredundant SAR202 MAGs (>50% completeness) revealed a substantial variation in their estimated genomic sizes (EGS) (1.1–5.1 Mbp) and GC content (29%–69%) (Fig. 1A; Table 1). Approximately 18% of SAR202 (*n* = 70) have small genome sizes (1.1–2 Mbp) and GC content below 40%, clustering within quadrant 1 (Fig. 1A). Other quadrants (2–4) are not considered gsSAR202 due to their larger genome sizes and/or higher GC content [20]. A few small SAR202 genomes have been reported in earlier studies. Two gsSAR202 with assembled genome sizes (70%–80% completeness) of 1.11 and 1.36 Mbp and low GC content (<40%) were found in the Arctic Ocean [14]. Two small genomes SAR202 (~1.36 Mbp, with 66%–68% completeness) were found in subgroups II and IV in the upper water column (<150 m) of the Caspian Sea [15], but their GC content is higher than 55%. Several small genomes of SAR202 (1.1–2 Mbp) were found in groups I, II, III, and V, but only one of them has a low GC content (<40%) [8]. Despite these scattered reports of small SAR202 genomes, genome streamlining in SAR202 has not been fully investigated. In this study, we identified 70 gsSAR202 from the current metagenome database.

**Table 1:** Genomic characteristics of SAR202 with small (<2 Mbp) and large genomes (>2 Mbp).

**SAR202 lineages**	**Estimated genome size (Mbp)**	**GC (%)**	**N50**	**Proteo-rhodopsin**	**CDS**	**tRNA**	**Gene richness**	**Gene density (%)**
group Ia_S	1.4–1.9	31–38	50 107–103 748	Yes	1042–1348	23–39	0.15–0.16	89–94
group Ib	1.3–1.6	29–31	29 842–113 549	Yes	940–1537	14–37	0.14–0.15	91–95
group Ic	1.1–1.6	29–33	30 679–124 327	Yes	687–1476	12–31	0.14–0.16	91–95
group IIa	1.3–2.1	29–30	17 950–174 376	Yes	962–1901	17–44	0.14–0.16	84–94
group IIb_S	1.4–1.7	34–35	11 838–118 131	Yes	938–1435	30–34	0.16–0.16	88–94
group IIIb_S	1.6–1.8	37–40	25 019–279 619	Yes	1272–1722	28–50	0.15–0.18	84–94
group VI_S	1.4–1.6	32–33	14 085–71 664	Yes	691–1558	15–39	0.16–0.16	80–92
group Ia	2.0–3.6	44–58	12 256–1 308 759	No	1197–2791	19–48	0.16–0.21	76–86
group Id-e	2.0–3.8	55–65	23 222–150 751	No	1561–3297	25–47	0.17–0.20	79–87
group IIc-f	2.2–4.8	41–63	9031–106 960	No	1270–4343	21–66	0.16–0.23	79–90
group IIIa	2.3–5.0	50–61	18 823–120 903	No	1257–4221	15–50	0.19–0.23	77–87
group IIIc-d	1.5–3.4	41–64	11 555–71 707	No	955–2820	20–48	0.18–0.20	78–88
group IV	1.4–2.9	43–67	13 575–212 392	No	819–2430	16–51	0.14–0.17	72–88
group V	1.4–2.1	41–61	13 233–126 382	No	700–1921	18–48	0.16–0.23	74–90
group VII	1.5–3.5	41–69	12 710–99 075	No	1251–3178	17–49	0.16–0.20	78–88
Unclassified	1.4–3.1	40–65	29 842–113 549	No	1094–2723	20–45	0.17–0.20	80–90

The 70 gsSAR202 genomes that we identified are distributed to six SAR202 subgroups (Ia, Ib, Ic, IIa, IIb, and IIIb) and one SAR202 group (VI) (Fig. 1B). A relatively high number of gsSAR202 is derived from subgroups Ia, Ib, Ic, and IIa. Group III is usually considered to have relatively larger genome sizes and occupy the deep ocean [4, 8, 15]. We identified seven gsSAR202 MAGs from group IIIb. The smallest gsSAR202 was found in subgroup Ic, with an EGS of 1.1 Mbp and a GC content of 31% (Table 1). gsSAR202 in subgroups Ib and Ic and group VI tend to have relatively smaller genome sizes (1.1–1.6 Mbp) and lower GC content (29%–33%) compared to the gsSAR202 in other groups. Within SAR202 group Ia, the gsSAR202 Ia_S clade displayed smaller genome sizes (1.4–1.9 Mbp) and lower GC content (31%–38%) than the non-gsSAR202 Ia clade, which includes the cultured strain JH545. Overall, gsSAR202 bacteria have reduced gene number and gene richness and have higher gene density than the non-gsSAR202 (>2 Mb) (Table 1). These features resemble the genome streamlining of bacteria [53, 54], suggesting that gsSAR202 might occupy the euphotic water layers. Intense competition for nutrients and the demand for energy efficiency in the euphotic ocean impose selective pressures favoring bacteria with streamlined genomes [16, 20].

### Predominance of gsSAR202 within the SAR202 community in the photic zone

**Figure 2:**
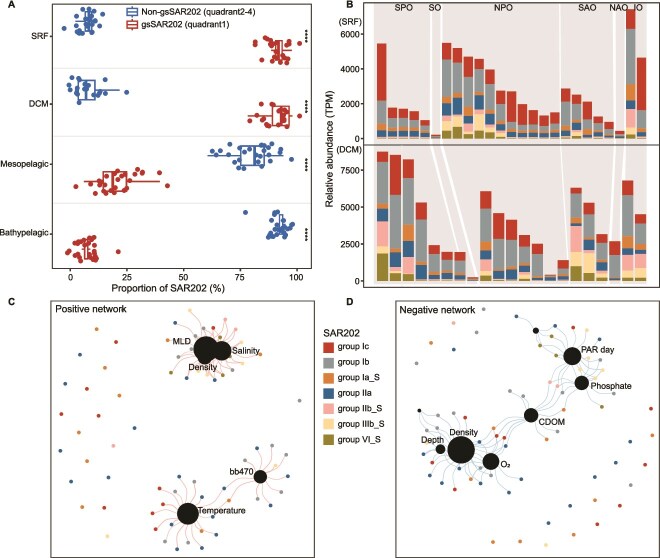
Distribution of gsSAR202 across typical oceanic water layers (SRF, DCM, Mesopelagic, and Bathypelagic). (A) Percentage of gsSAR202 in typical oceanic water layers. (B) Relative abundance (TPM) of various gsSAR202 clades in different oceans. (C) A positive correlation network between environmental factors and gsSAR202. D, a negative correlation network between environmental factors and gsSAR202. SRF, surface water; DCM, deep chlorophyll maximum; MLD, mixed layer depth; bb470, angular scattering coefficient, 470 nm; CDOM, color dissolved organic matter; PAR day, photosynthetically active per day.

The gsSAR202 within the SAR202 clade in the upper ocean dominated the SAR202 community (Supplementary Fig. S2A) and contained low GC content (Supplementary Fig. S2B) based on metagenomic data from BATS, Tara Oceans, and Maraspina. Our study revealed that the average abundance of gsSAR202 bacteria (3450 TPM) is significantly higher than that of non-gsSAR202 bacteria (364 TPM) in euphotic ocean regions (Wilcoxon, *P* < .001). The vertical profile of SAR202 bacteria in the ocean has been reported based on fluorescent in situ hybridization and metagenomics [3–5, 7, 8]. The current perception is that the relative abundance of SAR202 in the upper ocean is very low (<1%–2%), but they can make up 10%–20% of prokaryotic communities in the mesopelagic and bathypelagic zones. However, it has been reported that the absolute abundance of SAR202 in the upper ocean is higher than in the deep ocean [4]. The vertical profile of GC content shows that SAR202 bacteria in the euphotic zone have relatively low GC content (Supplementary Fig. S2B). A reduced GC content may facilitate genome streamlining by enhancing overall genomic efficiency. Specifically, AT-rich genomes require less energy for nucleotide synthesis because they predominantly utilize ATP and TTP, thereby diminishing the demand for GTP and CTP production [55]. Moreover, ATP is the most abundant nucleotide in the cytoplasm or within host cells, and this nucleotide bias likely promotes genome streamlining. On average, >90% of SAR202 on the surface and the DCM depth is composed of gsSAR202 (Fig. 2A), suggesting that the upper ocean is an important niche for gsSAR202.

Although gsSAR202 bacteria dominate the SAR202 community in the euphotic ocean, non-gsSAR202 bacteria with larger genome sizes and higher GC content also exist in the upper ocean. Non-gsSAR202 can contribute ~10% of SAR202 in the euphotic zone (<250-m depth) (Fig. 2A). The culturable SAR202 bacteria, obtained from the upper ocean, have a genome size of 3.1 Mbp and a GC content of 51.8% [13]. gsSAR202 bacteria appear to have simplified metabolic processes through reducing gene number and diversity, compared to non-gsSAR202 (Table 1). It is unclear how the genome streamlining of gsSAR202 affects our efficiency in cultivating them in the laboratory. SAR202 bacteria have drawn great attention lately due to their unique role in transforming RDOM in the deep ocean [10]. However, we know very little about the evolution and metabolic potential of these genome-streamlined SAR202 in the euphotic ocean.

### Distribution of gsSAR202 in the euphotic zone

To explore the distribution of gsSAR202 in the euphotic ocean, we analyzed the presence of different gsSAR202 clades and their relationship with environmental factors using surface and DCM samples from the Tara Ocean project. The gsSAR202 clades exhibited an uneven distribution across the euphotic zone of the ocean (Fig. 2B). Specifically, the highest abundance was observed in the Indian Ocean (7421 TPM), and the lowest was in the Southern Ocean (217 TPM). More than 50% of gsSAR202 are in groups Ib and Ic (Fig. 2B). Previous research indicates that the SAR202 community is vertically stratified, with subgroups such as Ib and Ic being more prevalent in the photic zone [4, 9]. To investigate the relationship between 70 gsSAR202 bacteria and 41 environmental factors, we conducted positive correlation (*r* > 0.3, *P* < .05) and negative correlation (*r* < −0.3, *P* < .05) network using Spearman’s correlation coefficients, which revealed generally weak correlations between SAR202 and environmental factors. The mixed layer depth (*r* = 0.49), temperature (*r* = 0.38), and the optical backscattering coefficient at 470 nm (bb470) (*r* = 0.43) are positively correlated with gsSAR202 (Fig. 2C). Because bb470 is influenced by particulate density in the water column, including high particulate organic matter, which can modulate light penetration, this suggests that nutrient availability, temperature, and light dynamics may all play roles in shaping gsSAR202 distribution. The mutational bias hypothesis proposes that higher temperatures reduce genome size by promoting the loss of nonessential DNA, whereas colder environments support larger genomes by slowing mutation rates and preserving noncoding or redundant DNA [56, 57]. gsSAR202 bacteria show negative correlations with density (*r* = −0.39), depth (*r* = −0.38), O_2_ (*r* = −0.32), fluorescent dissolved organic matter (*r* = −0.37), phosphate (*r* = −0.36), and photosynthetically active radiation (PAR) (*r* = −0.44) (Fig. 2D).

### Independent evolution of gsSAR202

**Figure 3:**
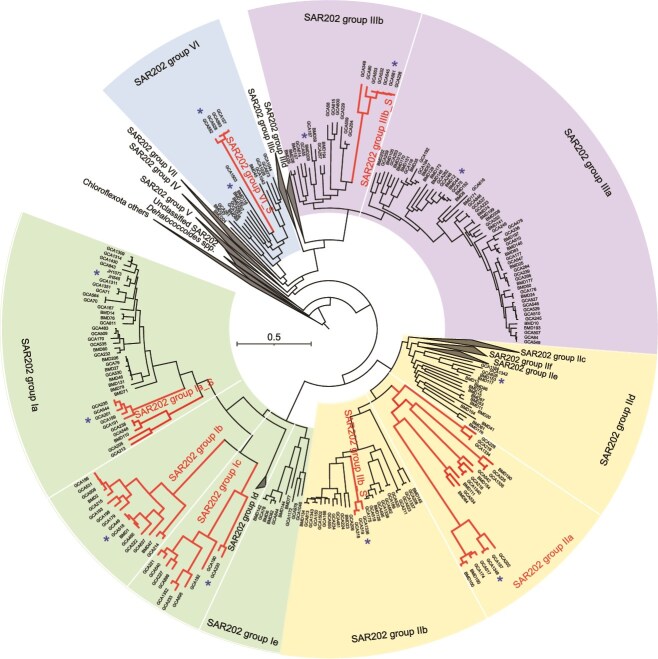
Phylogenomic tree based on 393 SAR202 bacterial genomes from seven major groups (I–VII). The branches with small SAR202 genomes are signed with subgroup labels. The representative genomes used for later analysis (Table 2) were labeled with *. For each clade, one representative was selected based on high completeness, low contamination, and average ocean abundance for Table 2 analysis. Bootstrap values greater than 50 in each branch. The clade nomenclature is consistent with previous studies [3, 4].

The phylogenomic analysis of gsSAR202 showed that they are distributed into four large SAR202 groups (I, II, III, and VI) among the total seven groups, but gsSAR202 genomes form their own phyletic lineages within the major groups (Fig. 3). The gsSAR202 subgroups Ia_S, Ib, Ic, IIa, IIIb_S, and VI_S follow independent evolutionary trajectories parallel to other SAR202 lineages. Divergence time estimates based on r8s analysis (Supplementary Fig. S4) indicate that the gsSAR202 branches in subgroups Ia_S and Ib diverged around 394 MYA during the Devonian period, later than other gsSAR202 clades such as IIa (555 MYA), IIb (567 MYA), and IIIb_S (457 MYA). The recently diverged cluster of gsSAR202 in group I dominates the SAR202 community in the euphotic zone, indicating strong adaptive evolution to upper-ocean conditions. In contrast, SAR202 group IIIa, which dominates in the dark ocean, represents a recent divergent cluster of non-gsSAR202 (276 MYA), suggesting adaptation to deep-ocean environments. The gsSAR202 occurs in Group III, which is generally considered dominant in the deep ocean. Recently, we reported that the number of genes encoded by subgroup IIIb varies greatly [9], suggesting their high genomic plasticity. Moreover, the predominance of subgroup IIIb in the upper ocean has been reported [4, 9]. These observations support the presence of gsSAR202 in subgroup IIIb. A subset of SAR202 in subgroup IIIb appears to have adapted to the upper ocean, undergoing genome reduction early in bacterial evolution. Despite their genome streamlining, gsSAR202 are still grouped with large genome SAR202 within the same subgroup or groups, suggesting that the core genes are still shared between gsSAR202 and large genome SAR202. The phylogenomic position of gsSAR202 is consistent with the phylogenetic tree based on the 16S rRNA gene sequences (Supplementary Fig. S3). The Devonian period (~419–359 MYA) was characterized by profound ecological transformations. This era witnessed the colonization of terrestrial habitats by vascular plants, significant fluctuations in atmospheric oxygen levels, and the emergence of intricate symbiotic relationships. These developments generated ecological niches and imposed selective pressures—such as variations in light availability and the establishment of oligotrophic conditions—that possibly promoted metabolic innovations and bacterial diversification, including the divergence of the gsSAR202 Ia_S and Ib lineages in the ocean.

*Chloroflexota* is an ancient bacterial group that occurred over 2 billion years ago [58]. Within the *Chloroflexota* phylum, SAR202 bacteria diverged from *Dehalococcoides* approximately 902 MYA during the Tonian period (Supplementary Fig. S4). *Dehalococcoides mccartyi* was a strict anaerobe strain and had an S-layer protein subunit cell wall lacking peptidoglycan [59]. The Tonian period, which is a precursor to the Cryogenian “oxygenation event,” was characterized by a gradual increase in atmospheric and oceanic oxygen levels [60]. This incremental oxygenation likely drove the evolution of aerobic respiration, which provides greater energy efficiency compared to anaerobic pathways. Additionally, during the Tonian era, eukaryotic lineages—such as early protists and algae—underwent significant diversification, creating new ecological niches and opportunities for bacterial adaptation as participants in nutrient cycles [60]. The availability of more complex nutrients and rising oxygen levels have probably driven the divergence of aerobic SAR202 bacteria from their anaerobic *Dehalococcoides* ancestors during this period. The subsequent evolution of gsSAR202 likely represents an adaptation to the contemporary oceanic environment.

### Metabolic characteristics of gsSAR202

To further understand the metabolic capability of gsSAR202, we compared key functional annotations of seven gsSAR202 genomes with five non-gsSAR202 genomes and two SAR11 reference genomes (Table 2). These representative genomes were selected based on their high completeness (>90%), low contamination (<5%), and high abundance in the ocean. gsSAR202 and SAR11 share a similar number of ORF and the percent GC content, especially for those gsSAR202 with genome sizes ranging from 1.3 to 1.4 Mbp. SAR11 bacteria contain more signal transduction genes than gsSAR202 (Table 2). SAR11 bacteria also contain about twice as many membrane transport genes and cell growth and death genes than gsSAR202, suggesting that SAR11 has a more substantial potential for interacting with its surrounding seawater environment than gsSAR202. SAR11 bacteria dominate the euphotic ocean, accounting for ~25% of the microbial community in the upper ocean [16, 61]. SAR202 probably makes up 1%–2% or even less of bacterial counts in the surface ocean [62]. The glycan metabolism capacity of both small and large genome SAR202 is reduced considerably compared to SAR11 (Table 2). Specifically, SAR202 lacks genes for lipopolysaccharide (LPS) and peptidoglycan biosynthesis (Supplementary Table S2). LPS and peptidoglycan are crucial components of bacterial cell walls [63], and the reduction of glycan metabolic genes could be related to the absence of peptidoglycan cell walls in SAR202. Previous studies revealed that SAR202 bacteria lack the peptidoglycan cell wall structure [8, 10]. SAR202 bacteria seem to maintain certain archaeal properties, such as archaeal flagella (see the later section).

**Table 2:** Comparative functional classifications based on KEGG annotation of gsSAR202, non-gsSAR202, and SAR11 genomes.

	**GCA220 (Ic)**	**GCA518 (Ib)**	**GCA201 (Ia_S)**	**GCA1349 (IIa)**	**GCA1336 (IIb_S)**	**GCA552 (VI_S)**	**GCA561 (IIIb_S)**	**JH545 (Ia)**	**BMD153 (IId)**	**BMD182 (IV)**	**BMD209 (IIIb)**	**BMD44 (IIIa)**	**HTCC** **1040**	**HTCC** **1062**
		**gsSAR202**							**Non-gsSAR202**				**SAR11**	
**Characteristics**														
**Size (Mbp)**	1.6	1.3	1.4	1.4	1.5	1.6	1.7	3.1	4.0	1.8	3.1	3.1	1.3	1.3
**ORFs**	1476	1283	1361	1285	1435	1558	1626	2834	3612	1564	2554	2677	1369	1382
**GC (%)**	30.7	30.7	31.0	31.0	34.0	34.0	40.0	52.0	60.0	60.0	58.0	55.0	29.5	29.5
**Completeness (%)**	90	94	96	91	93	98	96	100	97	95	90	93	99	100
**Carbohydrate-Active enZYmes**														
GT	15	6	12	10	7	6	12	25	30	11	11	11	20	19
AA	2	3	1	1	3	2	7	4	2	2	11	20	2	2
CE	1	5	2	3	1	4	6	5	13	3	15	16	3	3
GH	6	7	6	1	3	4	4	24	95	4	6	9	5	5
**Metabolism**														
Carbohydrate metabolism	197	187	204	142	151	184	197	341	314	168	254	250	171	178
Energy metabolism	94	76	80	77	110	107	108	120	144	98	143	143	119	119
Nucleotide metabolism	41	45	50	41	54	44	51	61	62	48	56	52	54	50
Xenobiotics biodegradation	32	33	27	33	28	36	46	39	57	25	112	94	11	16
Cofactors and vitamins	88	96	95	84	91	93	128	124	141	99	148	169	90	93
Amino acid metabolism	171	152	165	160	159	178	178	204	291	154	226	269	173	176
Glycan metabolism	16	8	19	7	9	9	16	20	30	8	11	7	48	52
**Genetic Information Processing**														
Transcription	4	1	3	3	3	3	3	3	4	3	2	3	4	4
Translation	80	71	78	76	81	83	76	83	75	78	73	79	76	76
Folding, sorting, and degradation	29	25	35	32	37	35	41	35	44	36	41	40	31	32
Replication and repair	52	54	58	45	55	56	56	62	63	73	62	54	55	54
**Environmental Information Processing**														
Membrane transport	31	30	35	34	41	44	48	36	51	25	38	29	65	66
Signal transduction	25	18	19	18	26	23	25	55	41	26	35	36	31	35
**Cellular Processes**														
Cell growth and death	9	10	10	11	10	11	9	14	17	13	15	12	21	21
Cellular community	35	29	30	25	22	31	33	46	46	35	48	36	42	42

### Fewer genes for carbohydrate and aromatic degradation in gsSAR202

**Figure 4:**
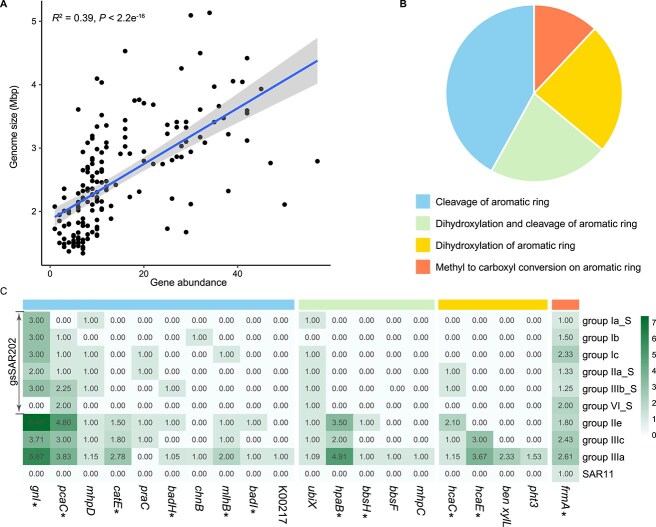
Analysis of aromatic compound degradation genes in SAR202 bacteria (only genomes with >85% completeness were considered). (A) Correlation between the number of degradation genes and the genomic size of SAR202. (B) Proportional distribution of degradation gene categories by reaction process. (C) The average number of degradation genes in SAR202 clades with small SAR202 genomes, large SAR202 genomes, and SAR11 genomes. A significant difference between gsSAR202 and Non-gsSAR202 was labeled with *. The full name and function of the genes are shown in Supplementary Table S4.

One of the well-known facts is that SAR202 bacteria in the deep ocean contain a large number of genes for degrading aromatic and polyaromatic organic compounds [4, 10, 64]. Here, we showed that the number of aromatic degradation genes in SAR202 decreases when genome sizes become smaller (Fig. 4A). Four major aromatic degradation genes (cleavage, methyl to carboxyl, dihydroxylation, and dihydroxylation and cleavage of aromatic ring) were identified (Fig. 4B). The comparison of 20 aromatic degradation genes between gsSAR202 and large genome SAR202 shows the reduction of gene numbers for gsSAR202 (Fig. 4C), suggesting that streamlining evolution led to the loss of many genes involved in complex DOM degradation. Regardless, gsSAR202 genomes contain some aromatic degradation genes whereas SAR11 has none of these genes (Fig. 4C), suggesting that gsSAR202 bacteria and SAR11 have different roles in the euphotic zone.

When compared with non-gsSAR202, gsSAR202 has a reduced number of the carbohydrate-active enzymes (CAZy) genes and the genes involved in central metabolism (carbohydrate, energy, amino acids, nucleotides, cofactors, and vitamins) and xenobiotic degradation (Table 2). The CAZy families break complex carbohydrates and glycoconjugates and allow cells to utilize complex carbohydrates [65]. The streamlined genomes such as gsSAR202 and SAR11 contain fewer CAZy genes than the large genome SAR202, implying that they use fewer carbon sources (Table 2). The SAR202 strain MD153 (IId), which has a large genome, contains the highest number of CAZy genes, including GH179, GH156, and GH33. In contrast, strains gsSAR202 GCA1349 (IIa) and GCA1336 (IIb_S) have fewer CAZy genes (Supplementary Fig. S5). Among these, GH179 enables to encode enzymes with β-N-acetylglucosaminidase activity, which hydrolyzes N-acetylglucosamine (GlcNAc) from glycoconjugates, glycoproteins, and glycolipids to release simpler molecules that microbes can further process [66]. Other gsSAR202 strains, gsSAR202 such as GCA220 (Ic), GCA518 (Ib), and GCA201 (Ia_S) have lesser CAZy genes compared to large-genome SAR202 BMD142 (Id) and cultured JH545 (Ia). Strain JH545 becomes more abundant in deep water (>200 m) and has a strong metabolism ability for sugar, such as L-fucose and L-rhamnose [13]. The presence of the CAZy genes in SAR202 genomes has been found in the Mariana Trench [8], which may enable SAR202 to utilize diverse carbohydrate sources such as starch, pectin, and chitin [6]. CAZy families like GH3, GH23, GH73, and GH103 are found exclusively in SAR11 bacteria (Supplementary Fig. S5), enabling them to process naturally abundant carbohydrates like cellulose, hemicellulose, glycoproteins, and peptidoglycan [67]. This enzymatic capability likely contributes to the dominance of SAR11 in the upper ocean by enabling them to utilize diverse organic carbon sources in oligotrophic environments.

### PR gene in gsSAR202

**Figure 5:**
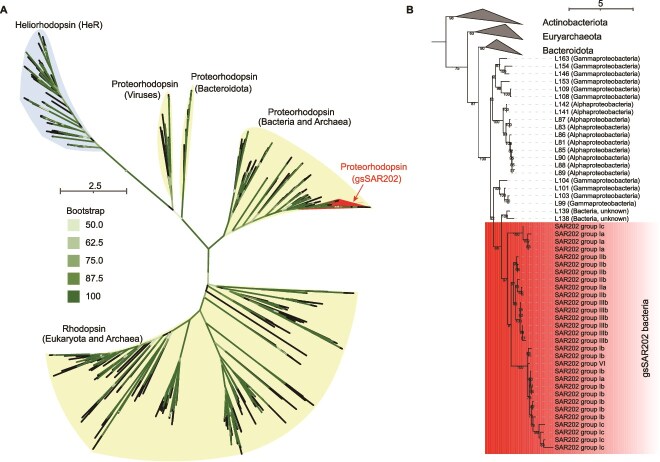
Phylogenetic tree based on amino acid sequences of the PR gene (A). The reference PR sequences from other organisms refer to Bulzu et al. All branches shown here have bootstrap value >60 (numbers not shown). A zoom-in tree of gsSAR202 is shown in (B). The alignment of conserved aligned domains is shown in Supplementary Fig. S4.

The PR gene was identified in all gsSAR202 groups through KEGG and eggNOG annotations, suggesting that it is widely distributed across gsSAR202. We constructed a phylogenetic tree based on PR genes from 30 gsSAR202 distributed across the seven streamlining SAR202 clades. The PR gene sequences in gsSAR202 are conserved and form a monophyletic lineage (Fig. 5A; Supplementary Fig. S6). Furthermore, subgroups of gsSAR202 cluster together based on similarities between the PR genes (Fig. 5B). SAR202 subgroups Ia_S, Ib, Ic, IIa, and IIb are known to contain the PR gene and occupy the surface water [4, 9], but the presence of PR in subgroup IIIb are reported here. The cultured SAR202 strain JH545, classified as non-gsSAR202 Ia, lacks the PR gene and exhibits growth inhibition under light exposure [13]. The PR gene is commonly present in bacterioplankton living in the sunlit zone of the oligotrophic ocean and provides energy support to a heterotrophic lifestyle [68, 69]. Two abundant bacterioplankton groups in the photic zone, SAR11 and SAR86, encode the PR gene and contain streamlined genomes [54, 70]. gsSAR202 bacteria share genome streamlining properties with SAR11 and SAR86, but they may adopt a different metabolic strategy in the euphotic zone.

### Various carbon compounds and nucleoside metabolism in gsSAR202

**Figure 6:**
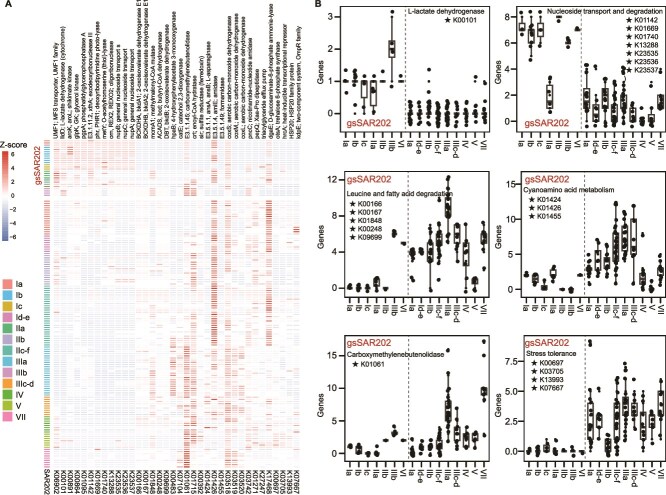
Significantly different metabolic profiles between gsSAR202 and non-gsSAR202 bacteria with edgeR package (parameters |logFC| > 2 and *P* < .05). (A) Heatmap of genes with significant differences between gsSAR202 and non-gsSAR202 bacteria. (B) Distribution of major metabolic processes in the SAR202 subgroups, based on different genes.

In comparison with non-gsSAR202, certain functional genes are significantly enriched in gsSAR202, whereas others are reduced (edgeR, |logFC| > 2 and *P* < .05) (Fig. 6A). The number of major facilitator superfamily (MFS) transporters (K06902) is more abundant in gsSAR202. The MFS family primarily facilitates the uptake of sugars, such as lactose and oligosaccharides [71, 72]. In the euphotic ocean, diverse sugars from primary productivity support microbial activity [73]. Genes such as L-lactate dehydrogenase and glycerol kinase are enriched in the gsSAR202 bacteria (Fig. 6A), suggesting a strong potential for sugar utilization. L-lactate dehydrogenase, a key enzyme in anaerobic glycolysis, is crucial for continuous ATP production under anoxic conditions [74]. L-lactate dehydrogenase gene is annotated in all gsSAR202 genomes but is nearly absent in the non-gsSAR202 (Fig. 6B), indicating that these bacteria can thrive in low-oxygen environments. The correlation analysis revealed that gsSAR202 negatively correlates with O_2_ concentration (*r* = −0.32) in the euphotic ocean (Fig. 2D), further supporting gsSAR202 thriving in the low O_2_ environment.

**Figure 7:**
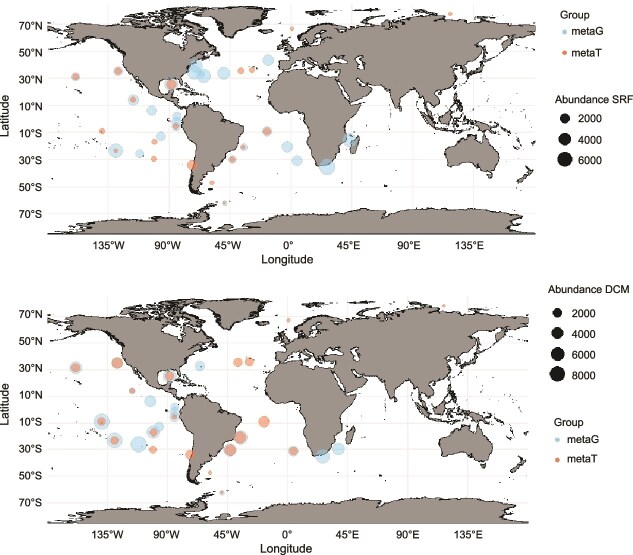
TPM abundance of gsSAR202 genes and transcripts in the surface (A) and DCM layers (B) of the global ocean. Data are derived from metagenomic and metatranscriptomic analyses. Point size reflects TPM abundance.

Nucleoside transport and metabolism genes are enriched in gsSAR202, except SAR202 IIa (Figs 6A and 7B). Specifically, nucleoside transport genes (*nupA*, *nupB*, and *nupC*), oligoribonuclease (*orn*), exodeoxyribonuclease (*xthA*), and deoxyribodipyrimidine (*phr*) are enriched in gsSAR202. Nucleoside transport genes facilitate the transport of nucleosides across cell membranes [75]. The capability to transport and recycle nucleosides efficiently could be important for the DNA and RNA synthesis of gsSAR202 bacteria. The orn gene encodes an exonuclease that degrades small oligoribonucleotides into mononucleotides, playing a crucial role in the complete degradation of nucleic acid fragments [76–78]. gsSAR202 bacteria appear highly competitive in scavenging oligonucleotides in the upper ocean. Alternatively, the sugar component of nucleotides and nucleosides can be utilized to produce oligosaccharides, which are important for glycosylation processes [79]. Such metabolic flexibility may relate to the ecological adaptation of bacteria in surface oligotrophic waters [80]. Furthermore, genes such as *xthA* and *phr* are enriched in gsSAR202 bacteria (Figs 6A and 7B) and are responsible for repairing UV-induced DNA damage [81]. The DNA-repairing mechanism might be important to surface-occupying gsSAR202, suggesting that light stress poses a challenge for non-gsSAR202 in the euphotic ocean. The only cultivated member of the non-gsSAR202 clade demonstrates sensitivity to light, exhibiting significantly slower growth under high light conditions [13]. This underscores the adaptations necessary for these surface-associated groups to counter sunlight-induced stress.

### Additional reduction and loss of genes in gsSAR202

The gsSAR202 has undergone genome reduction, losing a large number of coding genes to adapt to the euphotic marine environment. Specifically, mono/di-oxygenase families are more abundant in the non-gsSAR202 subgroups, particularly in SAR202 subgroup IIIa and group VII (Supplementary Fig. S7). Genes encoding catechol 2,3-dioxygenase (*catE*) and 4-hydroxyphenylacetate 3-monooxygenase (*hpaB*) are enriched in non-gsSAR202 (Fig. 6A; Supplementary Table S5). These genes have the potential to incorporate molecular oxygen into substrates, facilitating the breakdown and transformation of aromatic compounds by bacteria [82, 83]. Transcripts encoding catechol 2,3-dioxygenase in SAR202 bacteria are most abundant in the deep trench, indicating metabolic activity involved in the degradation of aromatic compounds within the deep ocean organic carbon pool [11]. Dehydrogenases, including 2-oxoisovalerate dehydrogenase and butyryl-CoA dehydrogenase, are absent in gsSAR202 but are rich in non-gsSAR202 (Fig. 6B). These genes play roles in the degradation of branched-chain amino acids such as leucine, valine, and isoleucine [84]. Additionally, genes coding for L-asparaginase (*ansA*, a*nsB*), amidase (*amiE*), and formamidase are more prevalent in non-gsSAR202 (Fig. 6B), enabling the utilization of various organic nitrogen forms [85, 86]. The carboxymethylenebutenolidase gene was enriched in non-gsSAR202 (Fig. 6B). This gene participates in the degradation of gamma-hexachlorocyclohexane and 1,4-dichlorobenzene [87], suggesting that non-gsSAR202 can utilize halogenated complex organic matter. A previous study also revealed that *Chloroflexi* MAGs have the potential to degrade halogenated complex organic matter in the sediment of the Mariana Trench [64].

Some genes that confer tolerance to extreme deep ocean environments are lost in gsSAR202 bacteria but are significantly enriched in non-gsSAR202 bacteria (Fig. 6B). These include genes associated with environmental stress tolerance, such as trehalose 6-phosphate synthase (OtsA), heat-inducible transcriptional repressor (HrcA), HSP20 family protein (HSP20), and OmpR family protein (KdpE). Trehalose 6-phosphate synthase (OtsA) is critical in trehalose biosynthesis and is crucial for bacterial survival under osmotic stress and cold conditions [88, 89]. HrcA maintains cellular homeostasis by repressing heat shock protein expression under normal conditions [90]. HSP20 is a crucial factor for organisms’ survival under sudden temperature increases or stress, as it protects cellular proteins from denaturation and aggregation [91]. SAR202 bacteria with smaller genome sizes in quadrant 2 (Fig. 1A) exhibit enrichment of genes encoding proteins likely involved in tolerance to extreme deep-ocean conditions, highlighting their adaptation to such conditions and differentiating them from gsSAR202 bacteria (Supplementary Fig. S8).

The gsSAR202 bacteria have fewer genes compared to non-gsSAR202 bacteria in pathways related to carbohydrate metabolism, energy metabolism, cofactors and vitamins, amino acid metabolism, glycan metabolism, and signal transduction processes, as indicated by SAR202 bacteria with >90% completeness (Table 2). However, functional annotation reveals that gsSAR202 bacteria possess complete central carbon metabolism pathways (glycolysis, citrate cycle (TCA), the pentose phosphate pathway) and amino acid biosynthesis pathways, supporting their independent metabolic activity (Supplementary Fig. S9). gsSAR202 bacteria contain genes for synthesizing vitamins K2, B2, B5, B9, and B12 (Supplementary Fig. S9; Supplementary Table S6), although vitamin K2 and B12 synthesis genes are only found in clades Ib and IIIb_S. VB12 synthesis was thought to be restricted to SAR202 group III [8].

### Cell wall structure of SAR202

SAR202 bacteria lack peptidoglycan cell walls; instead, they contain S-layer cell walls [8, 10, 13]. Two S-layer genes, *slaA* and *slaB*, which enable the production of S-layer proteins A and B, key components of the S-layer cell wall, were found in this study (Supplementary Fig. S9). The cell wall structure of SAR202 bacteria contains large amounts of cell surface glycoproteins, which may play a major role in the cell wall similar to peptidoglycan in other bacteria. One study reported the presence of sialic acid synthesis genes in SAR202, suggesting their role in forming the S-layer [10]. Although SAR202 contains cell division genes (*ftsZ*, *ftsA*, *ftsH*, *ftsK*), it lacks the *ftsW* and *ftsI* genes, which encode transpeptidases involved in cross-linking peptidoglycan in the division septum [92]. Archaea also use the *ftsZ* gene as the central organizer of cell division and similarly lack the *ftsW* and *ftsI* genes [93]. Moreover, certain groups of SAR202, including cultured strains, harbor archaeal flagellar genes such as *flaI* and *flaH* [13]. Together, this suggests that SAR202 bacteria maintain their archaeal-like cell structure not only due to ancient divergence but also possibly as a result of horizontal gene transfer between the two domains.

### Correspondence of gsSAR202 transcriptome in the upper ocean

To determine whether gsSAR202 bacteria are active in the surface ocean, we analyzed the relative abundance (TPM) of gsSAR202 genes and transcripts in the samples collected from the surface and DCM layers of the Tara Ocean (Fig. 7). Among the 10 stations where both gsSAR202 gene and transcript data were available (overlap of orange and blue, Fig. 7A and B), all 20 samples (surface and DCM) show active gene expression of gsSAR202, suggesting that gsSAR202 bacteria are not only present but also metabolically active in the upper ocean. The abundance of gsSAR202 decreases at higher latitudes, indicating that gsSAR202 bacteria are more inclined to warmer ocean environments. In general, gsSAR202 bacteria are more abundant in the DCM layer than at the surface, both at the genomic and transcriptomic levels (Fig. 7). It is likely that higher DOM produced by phytoplankton at DCM supports higher active metabolic activities of gsSAR202.

Transcriptomic analysis confirmed the expression of aromatic degradation genes from gsSAR202 in the euphotic ocean (Supplementary Fig. S10). For example, flavin prenyltransferase (ubiX), involved in the decarboxylation of aromatic acids, is identified in the gsSAR202 transcriptomes, suggesting that gsSAR202 bacteria are still involved in degrading some complex (i.e. semilabile) DOM. These aromatic degradation genes from gsSAR202 show higher expression abundance in the DCM water layer or the pelagic zone (Supplementary Fig. S11). The gsSAR202 PR genes are transcribed in the upper ocean and show higher relative abundance in the surface ocean than in the DCM (Supplementary Fig. S12), indicating that gsSAR202 might benefit from the light energy in the surface ocean. Based on the current metatranscriptomic database, this study primarily relied on metagenomic data, and the metatranscriptomic study is mainly to prove that gsSAR202 bacteria are active in the photic zone.

## Conclusion

**Figure 8:**
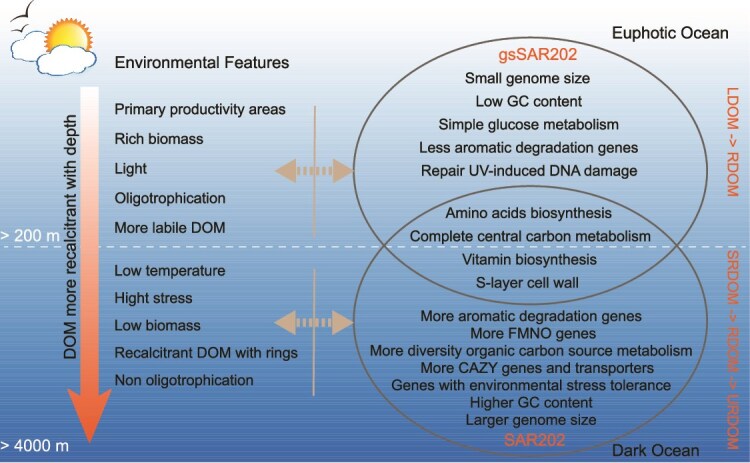
Overview of special niches and functions of gsSAR202 vs non-gsSAR202 in the ocean.

In this study, we reported the wide presence of genome-streamlined SAR202 in the euphotic ocean. gsSAR202 bacteria are found in several major lineages of SAR202, and they appear to have diverged from non-gsSAR202 later in their evolutionary history, evolving independently from larger-genome SAR202 bacteria. This divergence likely represents an adaptation to euphotic ocean conditions. Genomic analysis revealed that gsSAR202 bacteria harbor the PR gene and are enriched with UV-damaged repair genes, MSF transportation genes, and nucleoside metabolic genes (Fig. 8). These features enable gsSAR202 to adapt to the surface ocean and exploit diverse carbon sources, supporting their survival in nutrient-limited, sunlight-rich euphotic environments. Compared to non-gsSAR202, gsSAR202 genomes become streamlined, with reductions in genes associated with aromatic degradation, mono/di-oxygenase families, CAZy, and noncoding DNA. This genome streamlining reduces energy costs related to DNA maintenance and replication, facilitating adaptation to the oligotrophic surface ocean. Compared to SAR11, which has no aromatic degradation genes, gsSAR202 still contains a certain amount of aromatic degradation genes and possibly contributes to the utilization and transformation of labile and semi-labile DOM in the ocean. We also conducted the metatranscriptomic analysis and showed that gsSAR202 genes are actively expressed in the upper ocean. Even though the omics analysis unveiled the ubiquitous distribution of gsSAR202 in the surface ocean and their miniature nature, the biogeochemical role of gsSAR202 in the ocean deserves further studies. With the emergence of culturable non-gsSAR202 strains, cultivation of gsSAR202 will open the door to further understanding their biological and ecological functions.

## Supplementary Material

Supplement_words_wraf049

Supplement_Table_wraf049

## Data Availability

All SAR202 MAGs in the current study are available in the Figshare database (https://doi.org/10.6084/m9.figshare.26548255). For genomics analyses, shell scripts and data are used at Zenodo (https://doi.org/10.5281/zenodo.14252307). BATS metagenomes can be found on the NCBI (project no. PRJNA911943). All metagenomic and transcriptomic raw data and sample information are available in Supplementary Tables.
