# Supplementary material for: Genome-streamlined SAR202 bacteria are widely present and active in the euphotic ocean
Source: ISME J. 2025 Apr 8;19(1):wraf049. doi: 10.1093/ismejo/wraf049 (PMC11994032; doi:10.1093/ismejo/wraf049)
Supplement: Supplement_words_wraf049 [file supplement_words_wraf049.pdf]

Genome-streamlined SAR202 bacteria are widely present and active in the euphotic ocean

Short title: Active SAR202 in the Euphotic Ocean

Changfei He[1,2], Michael Gonsior[3], Jihua Liu[4], Nianzhi Jiao[1,*], Feng Chen[2,*]

[1] State Key Laboratory of Marine Environmental Science, College of Ocean and Earth Sciences, Carbon Neutral Innovation Research Center and Fujian Key Laboratory of Marine Carbon Sequestration, Xiamen University, Xiamen 361102, PR China

[2] Institute of Marine and Environmental Technology, University of Maryland Center for Environmental Science, Baltimore, Maryland 21202, USA

[3] Chesapeake Biological Laboratory, University of Maryland Center for Environmental Science, Solomons, Maryland 20685, USA

[4] Institute of Marine Science and Technology, Shandong University, Qingdao 266237, China

*Correspondence:

Feng Chen, Institute of Marine and Environmental Technology, University of Maryland Center for Environmental Science, 701 E Pratt Street, Baltimore, MD 21202, USA. Email: chenf@umces.edu;

Nianzhi Jiao, State Key Laboratory of Marine Environmental Science, College of Ocean and Earth Sciences, Carbon Neutral Innovation Research Center and Fujian Key Laboratory of Marine Carbon Sequestration, Xiamen University, A2-301, Zhou Long Quan Building, Xiang'an Campus, Xiamen University, Xiamen 361102, China. Email: jiao@xmu.edu.cn.

Supplement Figures

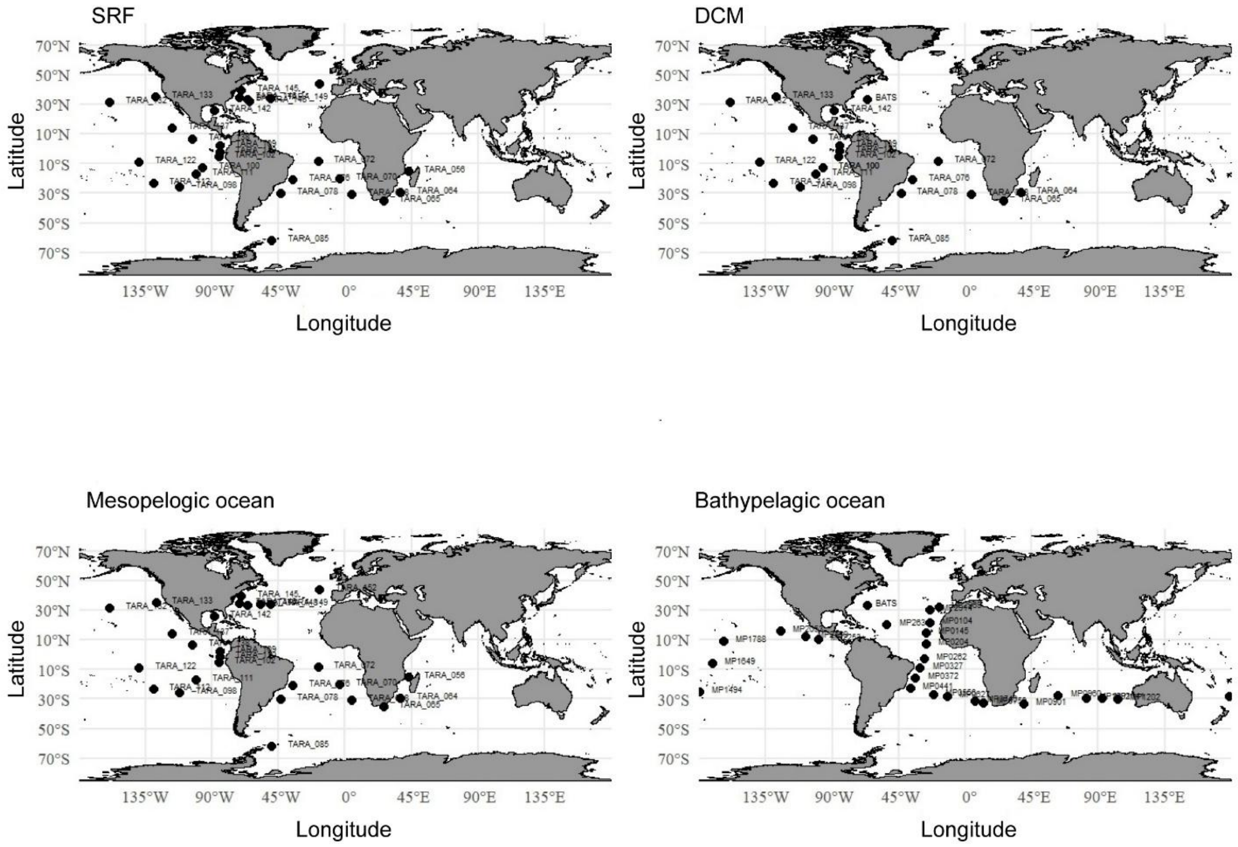

Figure S1. Station map. Surface ocean, SRF; Deep chlorophyll maximum, DCM.

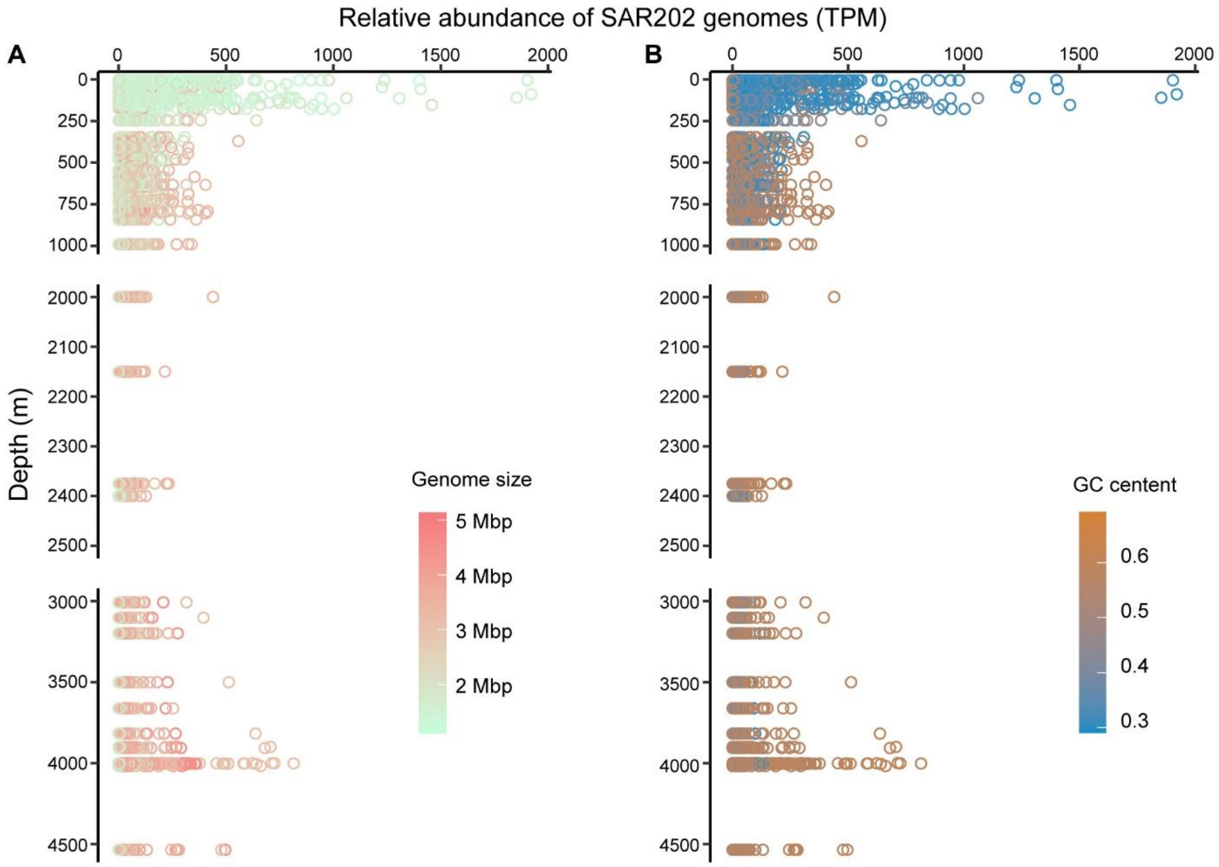

Figure S2. Vertical distribution of SAR202 bacteria present across ocean depths (0 to 4535 m) based on their TPM abundance. Vertical distribution of SAR202 bacteria present based on genome size (A) and GC content (B) of SAR202 bacteria (open circle).

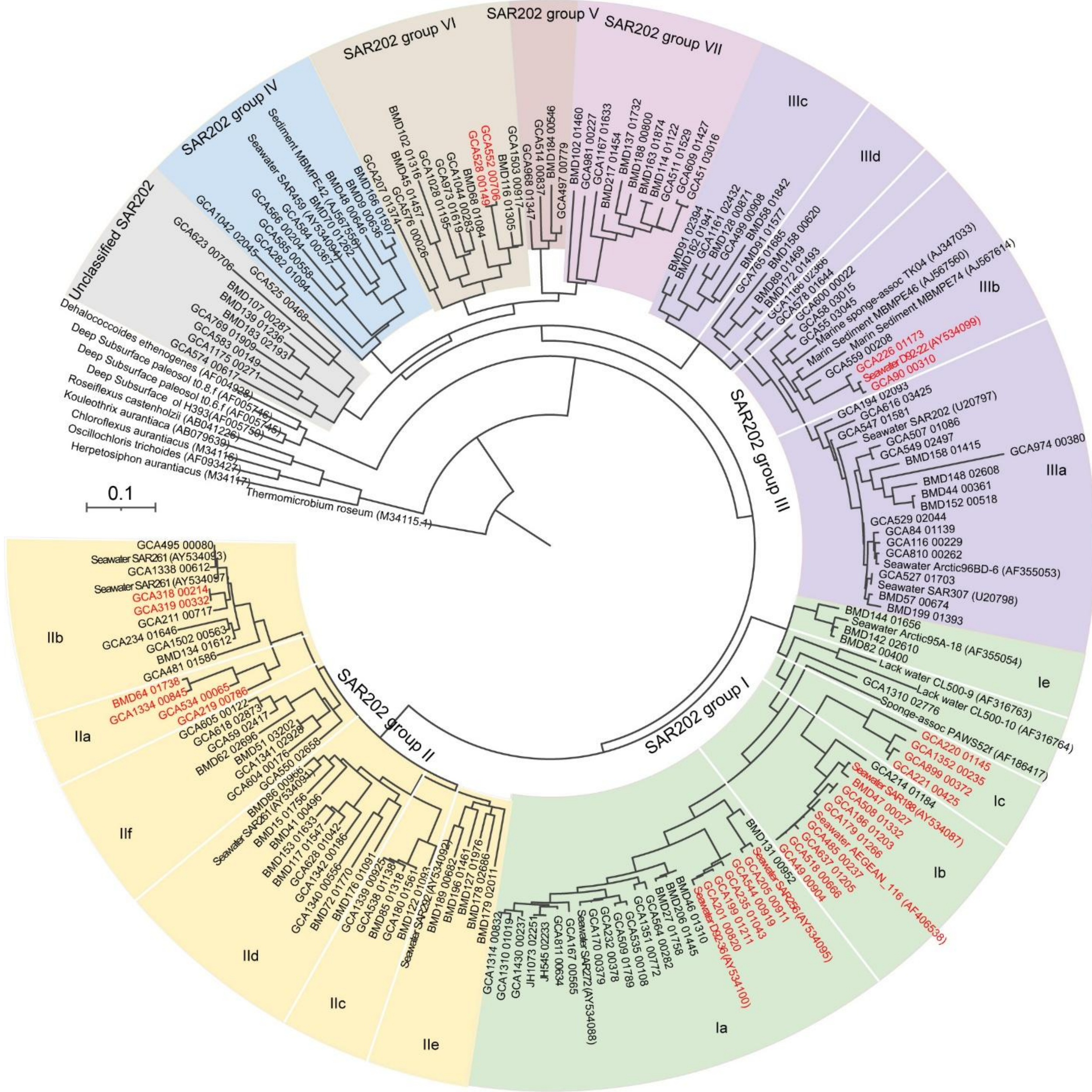

Figure S3. Phylogenetic tree of SAR202 bacteria based on 16S rRNA gene sequences. Bootstrap values based on 1000 replicates were more than 50% for all branches. Clades with small genome size and low GC content are highlighted with red labels.

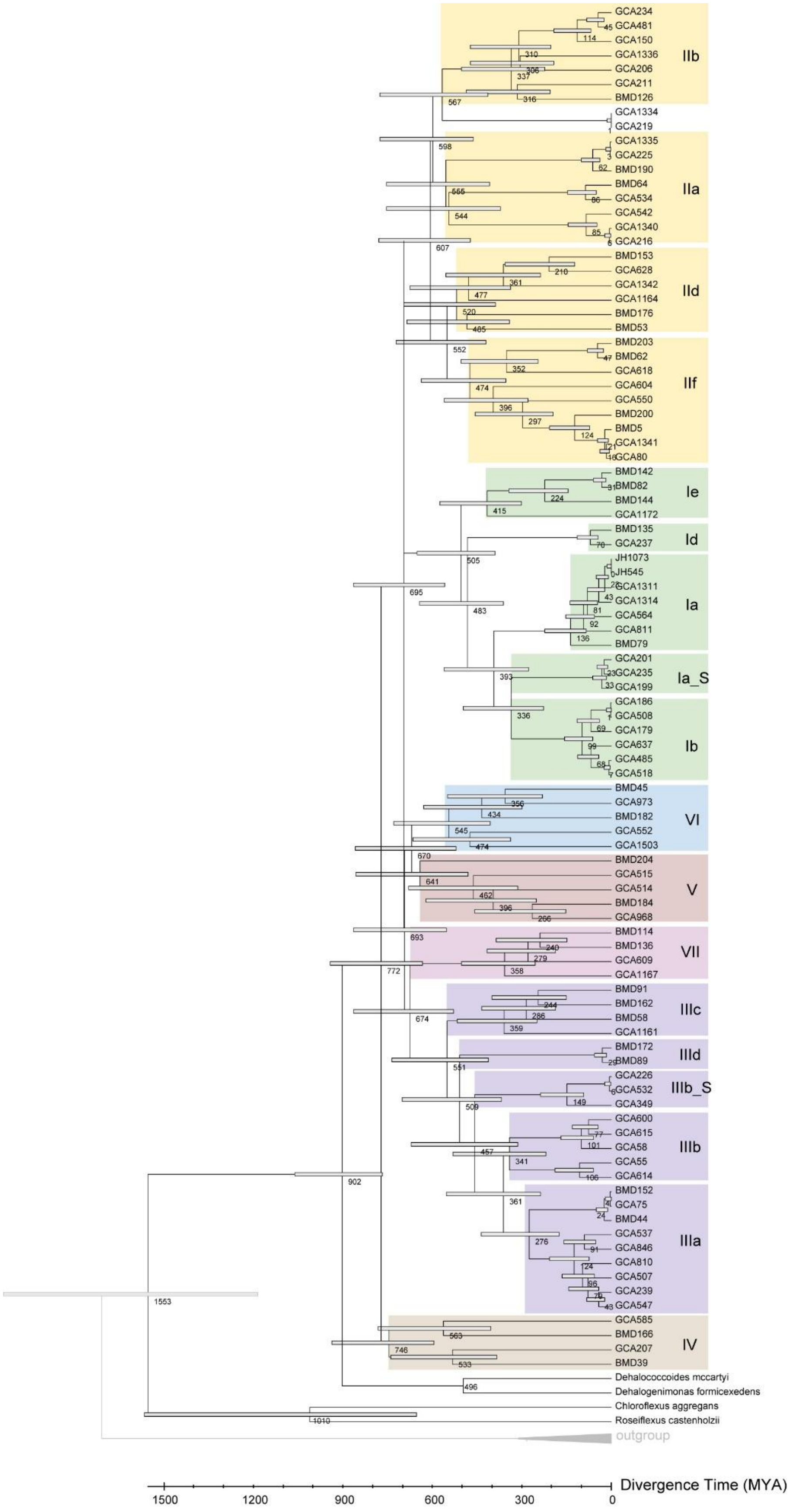
GCA234
GCA481
GCA150
GCA1336
GCA206
GCA211
BMD126
IIb
GCA1334
GCA219
GCA1335
GCA225
BMD190
BMD64
GCA534
GCA542
GCA1340
GCA216
IIa
BMD153
GCA628
GCA1342
GCA1164
BMD176
BMD53
IId
BMD203
BMD62
GCA618
GCA604
GCA550
BMD200
BMD5
GCA1341
GCA80
IIf
BMD142
BMD82
BMD144
GCA1172
Ie
BMD135
GCA237
Id
JH1073
JH545
GCA1311
GCA1314
GCA564
GCA811
BMD79
Ia
GCA201
GCA235
GCA199
Ia_S
GCA186
GCA508
GCA179
GCA637
GCA485
GCA518
Ib
BMD45
GCA973
BMD182
GCA552
GCA1503
VI
BMD204
GCA515
GCA514
BMD184
GCA968
V
BMD114
BMD136
GCA609
GCA1167
VII
BMD91
BMD162
BMD58
GCA1161
IIIc
BMD172
BMD89
IIId
GCA226
GCA532
GCA349
IIIb_S
GCA600
GCA615
GCA58
GCA55
GCA614
IIIb
BMD152
GCA75
BMD44
GCA537
GCA846
GCA810
GCA507
GCA239
GCA547
IIIa
GCA585
BMD166
GCA207
BMD39
IV
Dehalococcoides mccartyi
Dehalogenimonas formicexedens
Chloroflexus aggregans
Roseiflexus castenholzii
outgroup
1500
1200
900
600
300
0
Divergence Time (MYA)

Figure S4. The divergence time of SAR202 bacteria in the ocean. different color represents different SAR202 groups. The number is the divergence time in the clade nodes.

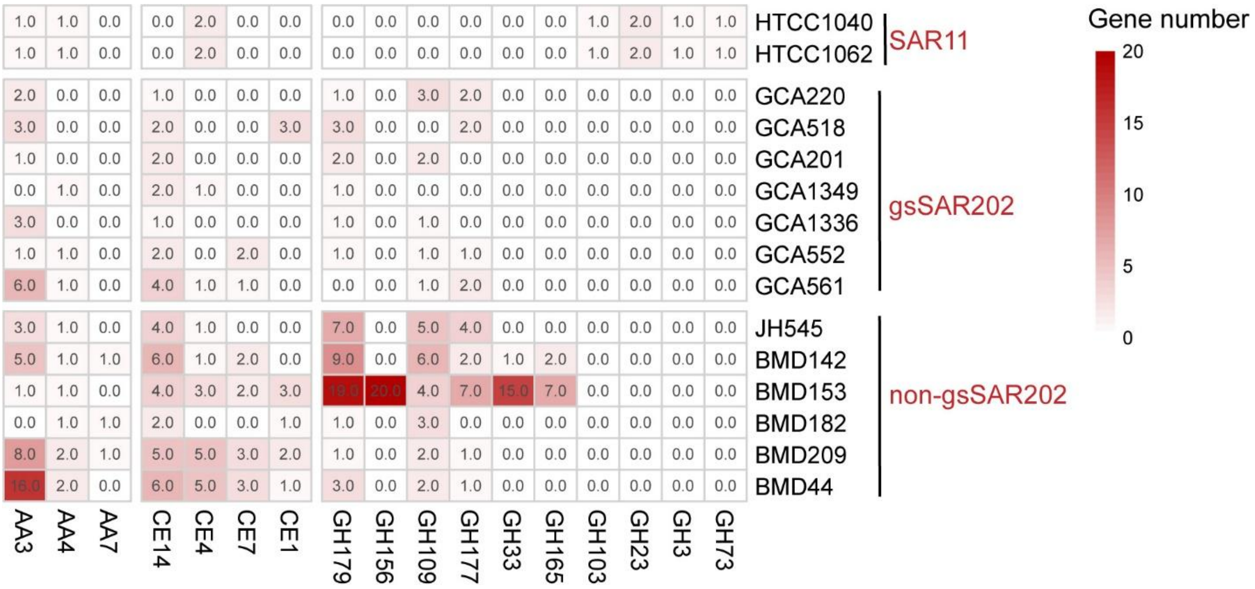

Figure S5. metabolic difference of the CZAY gene families between gsSAR202, non-gsSAR202 bacteria, and SAR11.

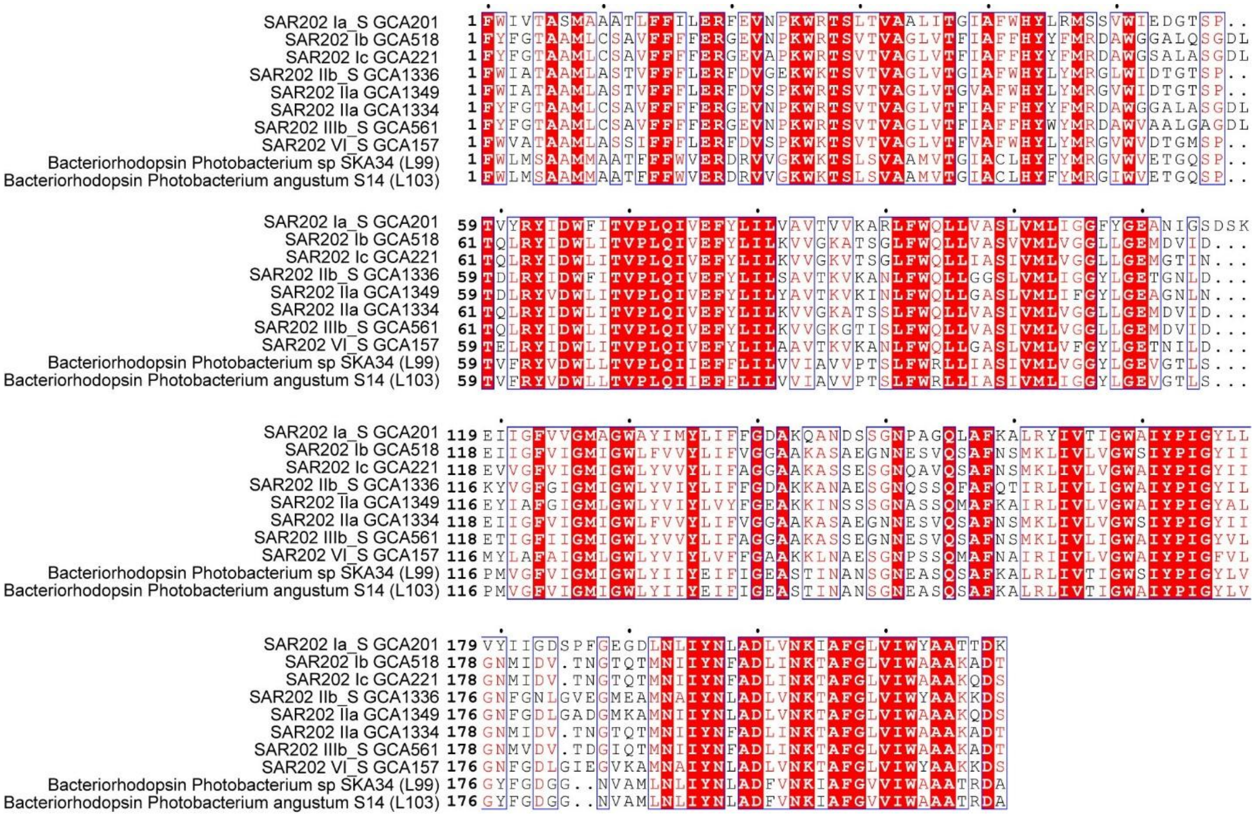

Figure S6. Conserved aligned domains of proteorhodopsin gene in the gsSAR202.

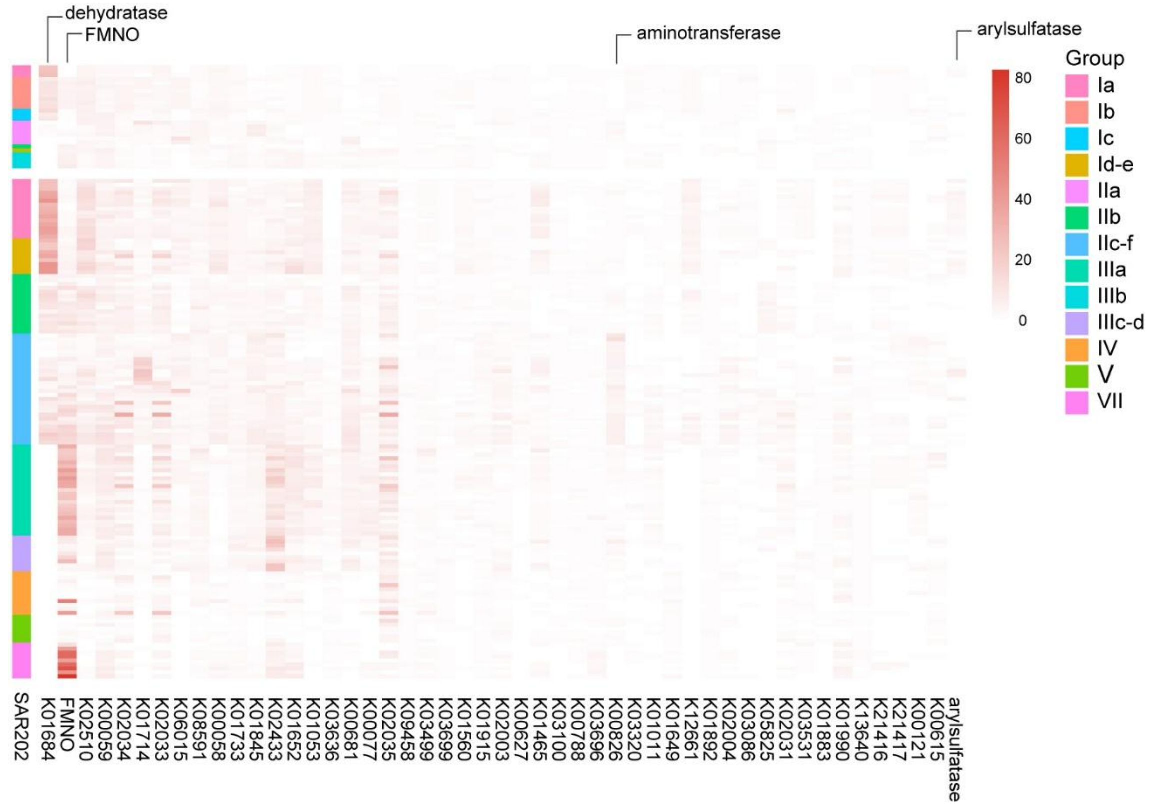

Figure S7. Heatmap of the most abundant KEGG categories in SAR202 genomes categorized by subgroups. The color bar indicates different SAR202 subgroups, with the blue-red gradient representing the variation of total number of genes.

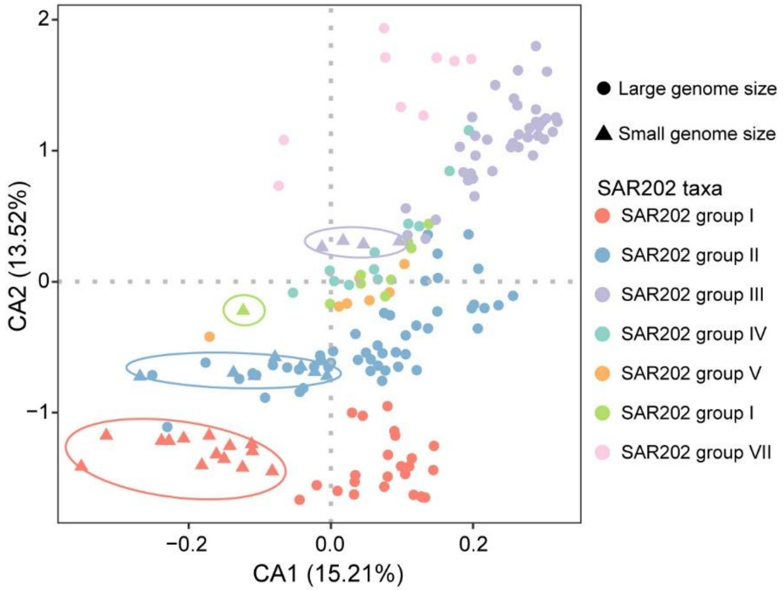

Figure S8. The genomic functional composition of various SAR202 bacteria based on KEGG annotation

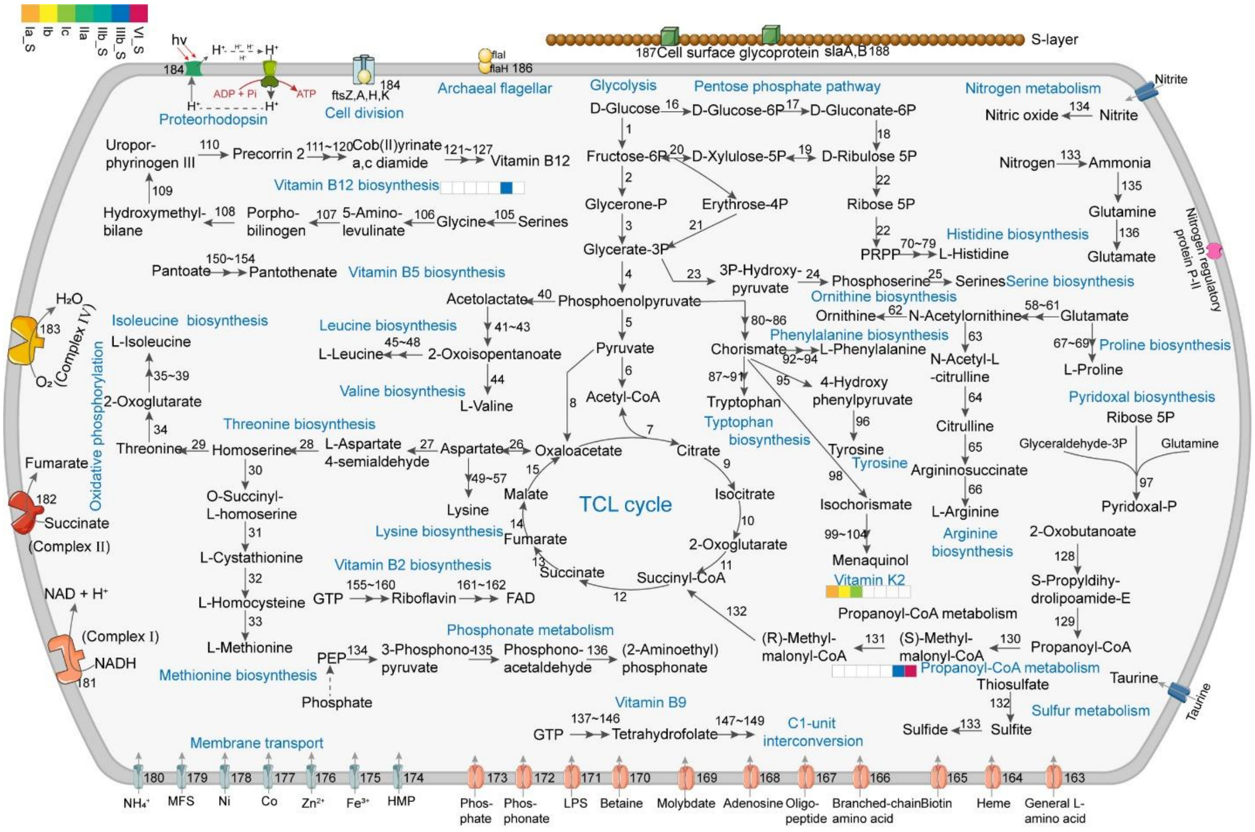

Figure S9. A proposed schematic view of the metabolic traits based on seven high-quality streamlined SAR202 bacteria from seven different clades. Key metabolic pathways are bold and highlighted in blue, and gene content is shown in Table S6. Metabolic pathways without color bars indicate their presence across all gsSAR202. White color bars signify the absence of the corresponding metabolic pathway.

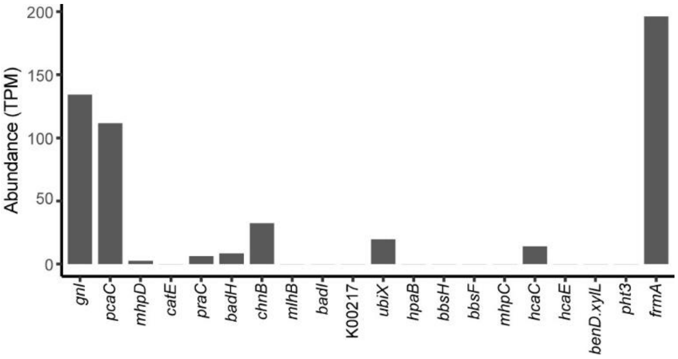

Figure S10. Abundance of aromatic degradation genes from gsSAR202 based on metatranscriptomic analysis.

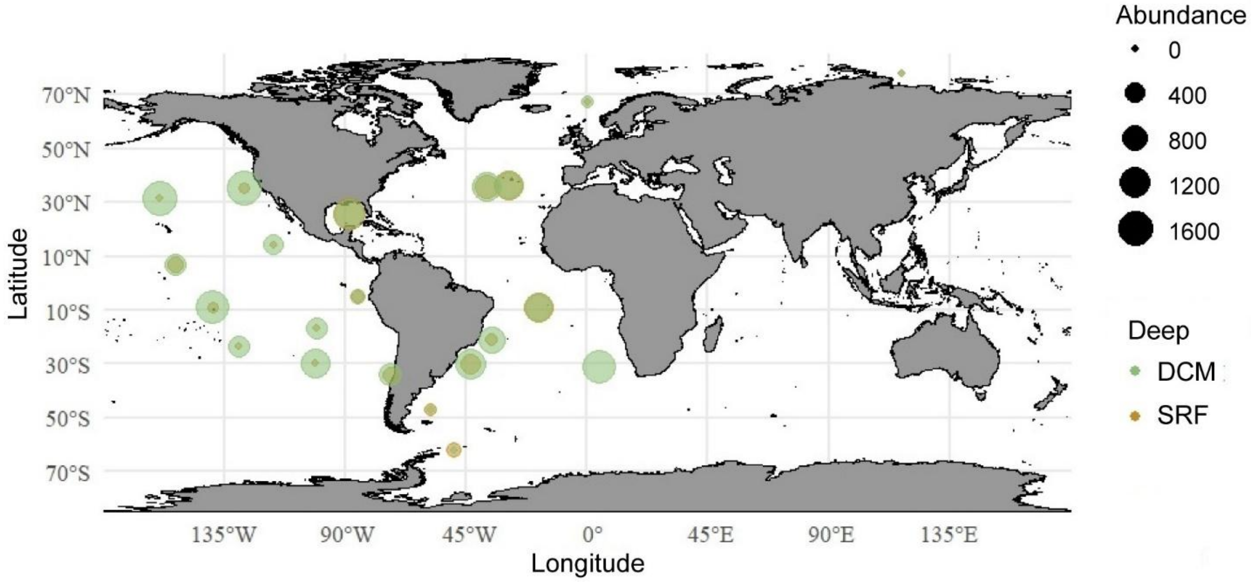

Figure S11. Distribution of aromatic degradation genes from gsSAR202 bacteria in the surface and deep chlorophyll maximum (DCM) layers of the ocean, based on metatranscriptomic data. Point size indicates the TPM abundance of these genes, with brown points representing gene abundance in the surface layer and green points indicating abundance in the DCM layer.

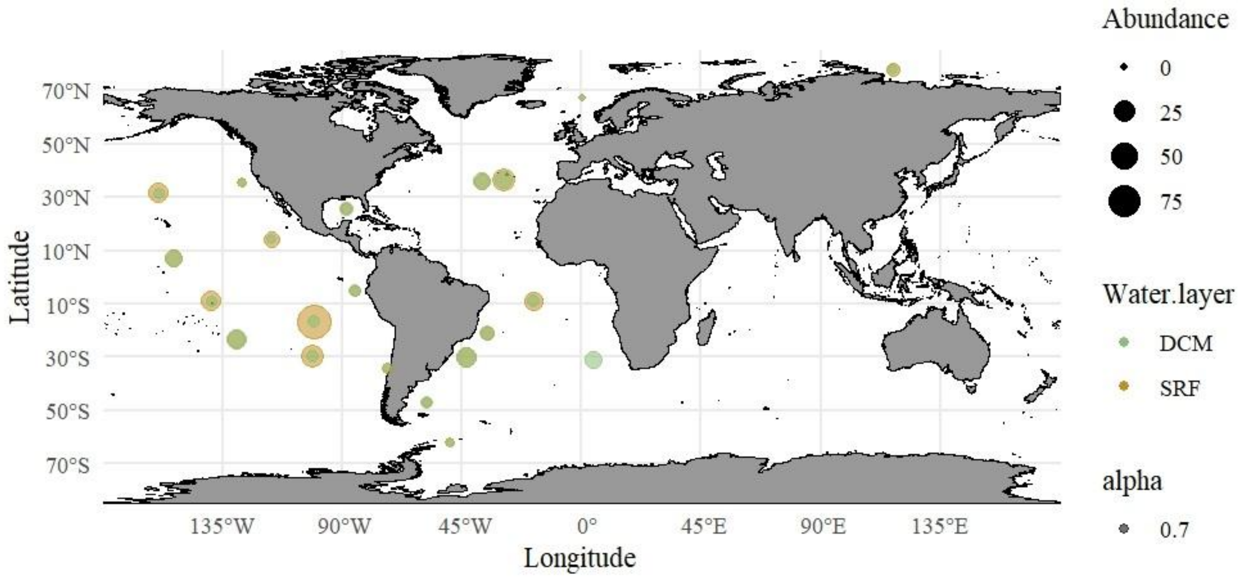

Figure S12. Distribution of Proteorhodopsin (PR) gene from gsSAR202 bacteria in the surface and deep chlorophyll maximum (DCM) layers of the ocean, based on metatranscriptomic data. Point size indicates the TPM abundance of these genes, with brown points representing gene abundance in the surface layer and green points indicating abundance in the DCM layer.
